# Supplementary material for: Chromothripsis during telomere crisis is independent of NHEJ, and consistent with a replicative origin
Source: Genome Res. 2019 May;29(5):737–49. doi: 10.1101/gr.240705.118 (PMC6499312; doi:10.1101/gr.240705.118)
Supplement: Supplemental Material [file supp_gr.240705.118_Supplemental_file_1.zip › contigs/annotated_contigs/DB78/contig.2.DB78_length_688_mean_cov_16.351744186.docx]

**DB78_length_688_mean_cov_16.351744186**

```
TGATGGAAGTATAAATGTGTAGGTGCAACATTTTGAAAGGCAGTCTAATAGTATCAGTGTGATTTTAGGTATTAATCTTAAGAAAATAA
 >chr15:66804280-66804597 + E=6e-179
TTCAAAAGTGTTCATTTGAATTGATGTCATCAATTGTTTATAATAGCAAAAAACTGGAAACTACTAAAATATCATATAATATGGAATTA

GTTATGTGACAAAAGAGCTGTGGTACTAGACTGCCAAAGAACATTGAATGACACAGACATTCATGATATAAAACTATTAGAATCCTGTT

TTTATTAGGCAAAATCCTATTAGATATCTAAATAGATACATTTCTATTTT|GA|AGGTTGGGCTCTTCAATGTTTTTTTGCACATGGTG
                                                     >chr15:66728354-66728723 + E=3e-21
TTCACTCCTGTGCCCATAATTTTTTTGTGTGCTGTTGCTTCTGTCTTTTCAAACAGTGCCCATTTTCTAGGTCCACCTCTTGTCTAGCT
0
TTTACCAGGTGCCCCAGCCCACACTGGTAACCACTGCATCCATGTTCTCTGACACTCGCTGTCTGTGGGATTTTTTTAGAAGTTCAACC

TTACTACGTAACTATTTAATGTGACTGTCACTTGTCTCCCCAGCTGGAACAAGGGAAAATGTTAGGATGACATAAGGTAGCACCTTAGA

CTTTGGCTTAGTCTCAACCACATCAGGCTCCTGAGCTCAAGGAAGAATCTTTTGGGCAGCCTAGGTT
```
